# Can photonic heterostructures provably outperform single-material geometries?

**DOI:** 10.1515/nanoph-2023-0606

**Published:** 2024-01-25

**Authors:** Alessio Amaolo, Pengning Chao, Thomas J. Maldonado, Sean Molesky, Alejandro W. Rodriguez

**Affiliations:** Department of Chemistry, Princeton University, Princeton, NJ 08544, USA; Department of Mathematics, Massachusetts Institute of Technology, Cambridge, MA 02139, USA; Department of Electrical and Computer Engineering, Princeton University, Princeton, NJ 08544, USA; Department of Engineering Physics, Polytechnique Montréal, Montréal, Québec H3T 1J4, Canada

**Keywords:** photonic bounds, heterostructure, inverse design

## Abstract

Recent advances in photonic optimization have enabled calculation of performance bounds for a wide range of electromagnetic objectives, albeit restricted to single-material systems. Motivated by growing theoretical interest and fabrication advances, we present a framework to bound the performance of photonic heterostructures and apply it to investigate maximum absorption characteristics of multilayer films and compact, free-form multi-material scatterers. Limits predict trends seen in topology-optimized geometries – often coming within factors of two of specific designs – and may be utilized in conjunction with inverse designs to predict when heterostructures are expected to outperform their optimal single-material counterparts.

## 1 Introduction

Photonic large-scale optimization or “inverse design” exploits variations of thousands to millions of structural degrees of freedom to maximize electromagnetic field objectives (e.g. absorbance, overlap with a known mode, or scattered power). This approach has begun to play an important role in the design of high-performing optical devices [1], leading to advances in nonlinear frequency conversion [2], multiplexing [3], and bandgap engineering [4]. While structural optimization is generally NP-hard [5], effectively forbidding guarantees of optimal solutions [1], a new, complementary, approach based on convex field relaxations is beginning to set tight constraints on performance in a variety of settings [5], [6], [7], [8], [9]. Examples of this method include recent limits on maximum angle-integrated absorption [10], scattering cross-sections [11], communication [12], field screening [6], dipole masking [6], local density of states [13], and non-linear frequency conversion [14], among other canonical electromagnetic objectives [7]. In addition to partially assessing the optimality of inverse designs, performance bounds can also yield insights into the physical processes that underpin desired wave behaviors. For instance, a bound that increases rapidly and then saturates beyond a certain characteristic length suggests a minimum device size needed to achieve a given phenomenon. Limit information can also be used to guide structural optimization toward optimal solutions [15].

However, existing formulations to calculate structure agnostic bounds has limited prior work to a single design medium against a background (vacuum) [7], precluding investigations of heterostructures comprising two or more design materials. Burgeoning use of these devices for ultrabroadband absorption [16], passive cooling [17], ultrafast photonics [18], [19], among other applications [20], and corresponding efforts to address fabrication challenges, highlight a growing need for definitive statements about the possible advantages of heterostructures. Potentially loose bounds and the NP-hardness of the inverse design problem mean that neither method on its own can prove heterostructure superiority. However, the two methods can be combined to provide performance certificates and therefore a new way of assessing the trade-offs associated with multi-material engineering.

In this article, we show that the dual-Lagrange formalism of Ref. [7] can be extended to handle an arbitrary number of design materials. We present two basic examples that showcase this theory: bounds on the maximum absorbed power from a plane-wave incident on a multilayer film or from an oscillating dipole in the vicinity of a free-form structure restricted to a square design region. Comparisons to topology-optimized designs show heterostructure bounds consistently coming within a factor of two of device performance. More fundamentally, we demonstrate heterostructure designs exhibiting greater performance than either of their corresponding single-material bounds, providing definitive proof that at least in these settings use of multiple materials is advantageous.

## 2 Formulation

The key idea underpinning recent bound optimizations, detailed in Refs. [7], [8], [9], [10], involves relaxing structural and physical information in the typical field optimization problem by reducing the vector field constraints stated by Maxwell’s equations to a user-defined set of scalar constraints ensuring the conservation of power over sub-volumes of the total design region – generalizations of Poynting’s theorem [7], [13]. Convex relaxations of the resulting quadratically constrained quadratic program via duality [5] or semi-definite programming [21], [22] provide a bound on the original (primal) objective. More precisely, optimization of a given quadratic field objective *f* over the possible polarization fields |*ψ*_*k*_⟩ that may arise from a set of harmonic fields |*S*_*k*_⟩ incident on a given design region *V* can be framed as a quadratic program of the form

(1) $$\begin{array}{cc} \underset{\left\{\psi_{k}\right\}}{\max}\; & f\left(\left\{|\psi_{k}〉\right\}\right)\\ \text{s.t.}\; & 〈S_{k}|P_{j}|\psi_{k^{\prime }}〉-〈\psi_{k}|\left(\chi_{k}^{-†}I-G_{0}^{\left(k\right)†}\right)P_{j}|\psi_{k^{\prime }}〉=0,\\ & \;\forall j,k,k^{\prime } \end{array}$$

where *χ*_*k*_ denotes the electric susceptibility of the design material at frequency *ω*_*k*_, and $G_{0}^{\left(k\right)}$ the corresponding vacuum propagator acting on sources to yield their respective fields in vacuum – namely, via convolution of the vacuum Green’s function $G_{0}^{\left(k\right)}\left(r,r^{\prime },\omega_{k}\right)$ satisfying $\frac{c^{2}}{\omega_{k}^{2}}\nabla \times \nabla \times G_{0}^{\left(k\right)}\left(r,r^{\prime },\omega_{k}\right)-G_{0}^{\left(k\right)}\left(r,r^{\prime },\omega_{k}\right)=\delta \left(r-r^{\prime }\right)$ . Here, I and $P_{j}$ represent spatial projections into either the full or a subset *V*_*j*_ ∈ *V* of the design region *V*, respectively, and bra-ket notation is used to express complex vector fields over *V*. The integral constraints in Eq. (1) enforce Poynting’s theorem – power conservation – over each selected region. The Lagrange dual of Eq. (1) bounds the primal objective [23].

Extending this formalism to multiple materials may be carried out as follows. Expressing |*ψ*_*k*_⟩ = ∑_*m*_|*ψ*_*k*,*m*_⟩ as a sum of polarization currents associated with a given material *m* of susceptibility *χ*_*k*,*m*_, it suffices to allow each region to be filled with any combination of the selected *χ*_*k*,*m*_. To avoid unphysical solutions, however, one must also enforce an additional constraint precluding the overlap of distinct materials (polarization currents associated with distinct materials must be orthogonal in any sub-region). Performing this modification, the optimization problem becomes:

(2) $$\begin{array}{cc} \underset{\left\{\psi_{k,m}\right\}}{\max}\; & f\left(\left\{|\psi_{k,m}〉\right\}\right)\\ \text{s.t.}\; & \underset{m}{\sum }\left(〈S_{k}|P_{j}|\psi_{k^{\prime },m}〉-〈\psi_{k,m}|\chi_{k,m}^{-†}P_{j}|\psi_{k^{\prime },m}〉\right.\\ \; & \left.-\underset{m^{\prime }}{\sum }〈\psi_{k,m}|-G_{0}^{\left(k\right)†}P_{j}|\psi_{k^{\prime },m^{\prime }}〉\right)=0\;\forall j,k,k^{\prime },\\ & 〈\psi_{k,m}|P_{j}|\psi_{k^{\prime },m^{\prime }}〉=0\;\forall j,k,k^{\prime },m\neq m^{\prime }. \end{array}$$

**Figure 1:**
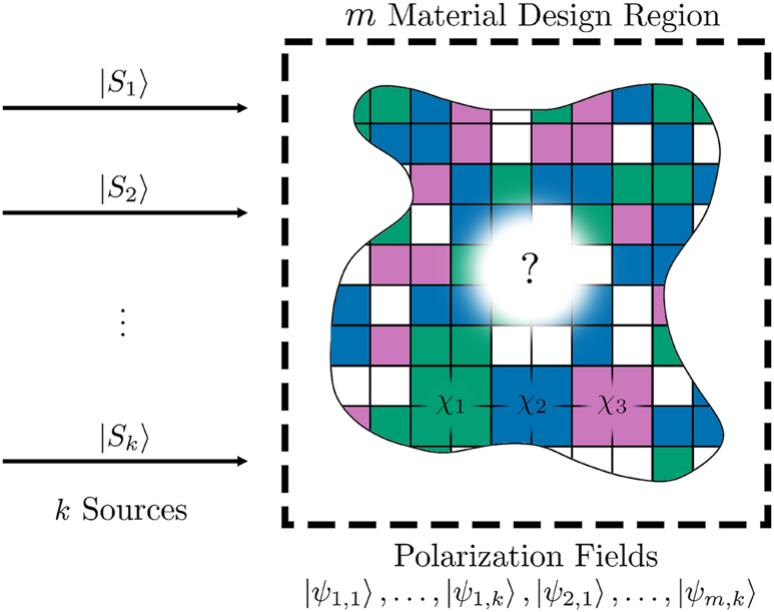
*k* sources are incident on an *m* material design region, and polarization currents break down into *k* × *m* components. Each computational voxel in the design region can be any of *m* materials (exclusively) or background. Objectives are written in terms of polarization currents.

A schematic of the problem under consideration is shown in Figure 1. Detailed calculations of the corresponding Lagrange dual and gradients, as well as a proof of the existence of dual solutions are provided in the Supplementary Material (SM) [24].

The preceding problems assume a design region containing vacuum as the background medium. If background is instead chosen to have susceptibility *χ*_*b*_ ≠ 0, defining |*ψ*_*k*,*b*_⟩ ≠ 0 as the background polarization current, a corresponding bound can be obtained from (2) via the following substitutions: $G_{0}^{\left(k\right)}\to G_{b}^{\left(k\right)}$ , |*S*_*k*_⟩ → |*S*_*k*,*b*_⟩, *ψ*_*k*,*m*_ → Δ*ψ*_*k*,*m*_, *χ*_*k*,*m*_ → Δ*χ*_*k*,*m*_. Here, $G_{b}^{\left(k\right)}$ is the operator form of the background Green’s function, satisfying $\frac{c^{2}}{\omega_{k}^{2}}\nabla \times \nabla \times G_{b}^{\left(k\right)}\left(r,r^{\prime },\omega_{k}\right)-\left(1+\chi_{b}P_{\Omega }\right)G_{b}^{\left(k\right)}\left(r,r^{\prime },\omega_{k}\right)=\delta \left(r-r^{\prime }\right)$ , where $P_{\Omega }$ projects to the entire design region, $|S_{k,b}〉=G_{b}^{\left(k\right)}{\left(G_{0}^{\left(k\right)}\right)}^{-1}|S_{k}〉$ , Δ*χ*_*k*,*m*_ ≡ *χ*_*k*,*m*_ − *χ*_*b*_, and |Δ*ψ*_*k*,*m*_⟩ ≡|*ψ*_*k*,*m*_⟩ − |*ψ*_*k*,*b*_⟩, Finally, the objective is reformulated in terms of |Δ*ψ*_*k*,*m*_⟩ as detailed in the SM.

The remainder of the article showcases the utility of Eq. (2) by considering bounds on two representative scattering problems: maximizing absorption from a planewave and a dipolar source. In these settings, the absorption objective can be written as

(3) $$f=\underset{k,m}{\sum }\frac{Zc}{2\omega_{k}}〈\psi_{k,m}|\frac{Im\chi_{k,m}}{{\left|\chi_{k,m}\right|}^{2}}|\psi_{k,m}〉,$$

with *Z* denoting the vacuum impedance. Resulting bounds are compared to the best performing devices obtained via topology optimization, as outlined in Ref. [25] for single materials and in the SM [24] for multiple materials. For a fair comparison between designs and bounds, inverse designs are only explicitly binarized in the case where no vacuum is permitted. In these cases, binarization was found to have a marginal effect on performance.

## 3 Planewave incident on a multilayer film

We first consider a TM (electric field out of the plane) planewave of wavelength *λ* incident on a device of length *L* ≤ *λ* consisting of multiple layers of variable thicknesses. We seek to maximize the ratio of absorbed to incident power. The computational ease of this effectively one dimensional problem allows us to readily enforce the constraints in Eq. (2) over each pixel of the computational grid. We first consider the use of either a single dielectric of *χ* = 4 + 0.1*i*, a single metal of *χ* = −4 + 1*i*, or of both materials with and without vacuum, henceforth referred to as the V and NV configurations, respectively.

**Figure 2:**
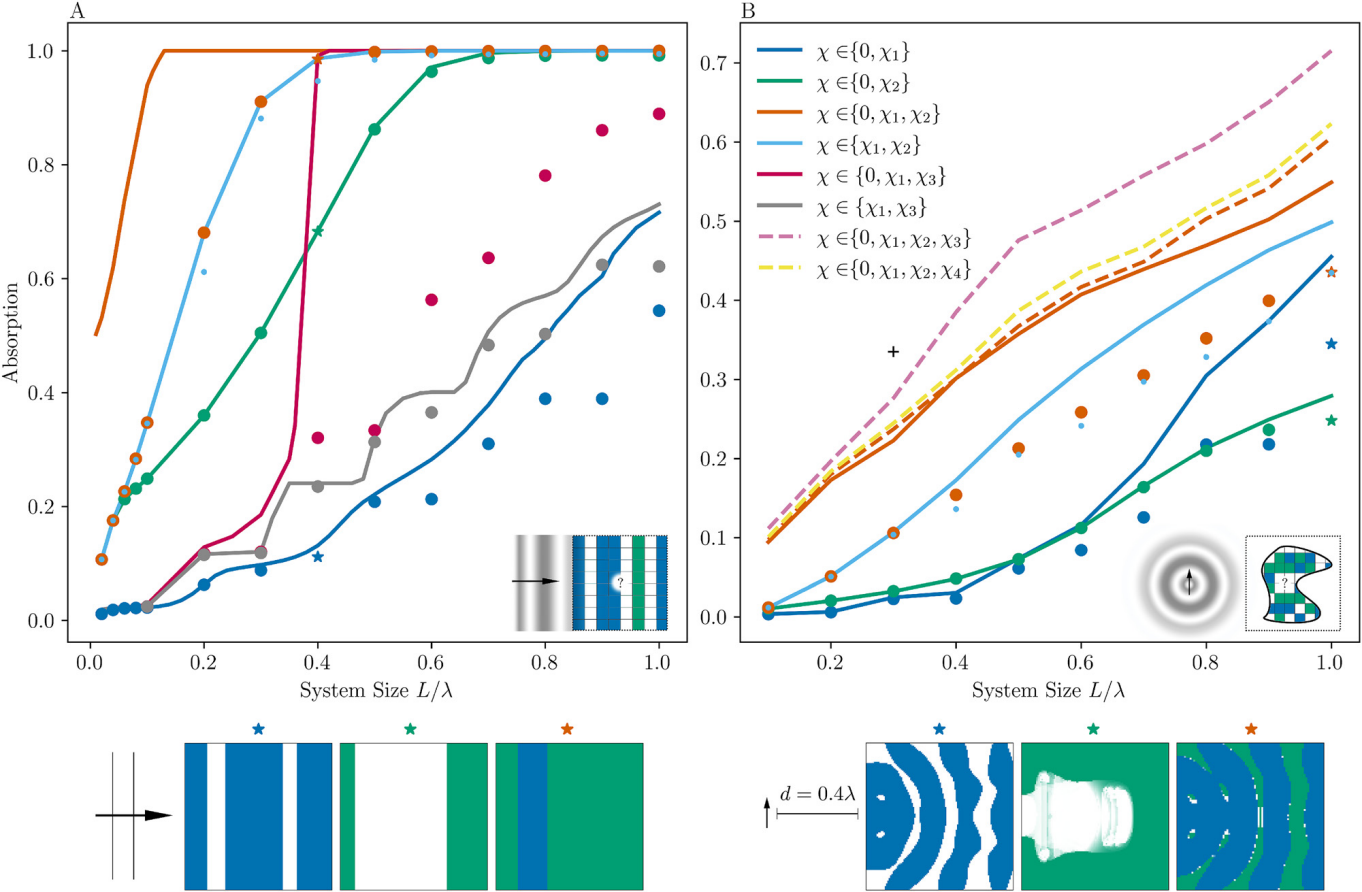
Bounds on maximum absorption (solid lines local constraints, dashed lines global constraints) and associated inverse designs (dots) for either a planewave incident on multilayer films (A) or an oscillating dipole near structures contained within a square design region (B). Absorption values quantify the net power absorbed in the respective design regions normalized to the incident power for the planewave or emitted power of the dipole in vacuum, for systems comprising various combinations of *χ*_1_ = 4 + 0.1*i*, *χ*_2_ = −4 + 1*i*, *χ*_3_ = 8 + 0.2*i*, *χ*_4_ = 8 + 1*i*, and vacuum regions. Representative inverse designs (*L* = 0.4*λ* for films and *L* = 1*λ* for compact structures) are labeled with stars and shown below the main plots. The colors representing each material are chosen to be the same for both inverse designs and bounds. Heterostruture designs outperforming single-material bounds, while context- and material-dependent, yield definitive proof of the utility of employing multiple materials (including vacuum).

Results are shown in Figure 2A and compared to topology-optimized designs. Notably, performance values for optimized structures and associated bounds reflect the increased ability of thicker films and greater material choice to achieve higher planewave absorption, which eventually saturates to unity absorptivity for sufficiently large devices. Note that all designs of size *L*/*λ* = 0.1 were found to be globally optimal via brute-force optimization and that absorption vanishes as *L*/*λ* → 0. Bounds are found to correctly anticipate the earlier onset of perfect absorbance and the provably superior performance of heterostructures compared to their single-material counterparts. This increased performance is evident at intermediate thicknesses 0.1*λ* ≤ *L* ≤ 0.5*λ* but rapidly vanishes in highly subwavelength or optically thick films, with metallic films outperforming their dielectric counterparts, and heterostructures primarily if not entirely composed of metal. As expected, metallic devices outperform dielectrics at small *L*/*λ* by exploiting plasmonic confinement, with dielectrics becoming increasingly effective as *L* → *λ*. Optimized designs suggest that the primary mechanism by which hybrid heterostructures outperform single-material structures at intermediate *L*/*λ* is by the creation of metallic cavities filled with dielectric (as opposed to vacuum) absorbing material. We note the non-additivity of the bounds: calculated limits on heterostructure absorbance are not given by the sum of the single material limits. In fact, topology optimized heterostructures outperform the sum of the two single-material bounds when 0.08*λ* ≤ *L* ≤ 0.5.

As seen from the optimized multilayer structure of thickness *L* = 0.4*λ* shown on the inset (orange star), there appears to be little to no use of vacuum when both metals and dielectrics are available. Indeed, while the bounds suggest that incorporating vacuum regions yields significant performance improvements, the inverse designs do not. This is unsurprising from the perspective of *effective medium theory*: for sufficiently sub-wavelength structuring, the metal-dielectric systems can respond like any intermediate dielectric (including vacuum). In contrast, for heterostructures composed of dielectrics alone, the introduction of vacuum represents a new otherwise unavailable medium. Consequently, the bounds and inverse designs for *χ* ∈ {4 + 0.1*i*, 8 + 0.2*i*} demonstrate a provable advantage in device performance for V over NV structures (Figure 2A).

## 4 Dipole near a compact structure

Next, we consider a TM-polarized dipole oscillating at frequency *λ*/*c* and located a distance *d* = 0.4*λ* from a square design region of side length *L* ≤ *λ*. We now seek to maximize the ratio of absorbed power to the power radiated by the dipole in vacuum, again enforcing constraints over each computational pixel. We consider the use of a dielectric with *χ* = 4 + 0.1*i*, a metal with *χ* = −4 + 1*i*, and the use of both materials with and without vacuum.

Results are shown in Figure 2B and compared to topology-optimized designs. Similar to the multilayer examples, bounds are seen to accurately predict trends in device performance, with the non-additive nature of multiple-material bounds becoming particularly pronounced for wavelength-scale devices. The performance of two-material devices is better than any single-material structure for 0.1*λ* ≤ *L* ≤ 0.9*λ*. Inspecting bound solutions and inverse designs, we postulate that the dielectric (blue star) and metallic device (green star) of size *L*/*λ* = 1 work by reflecting incident fields into an absorbing block near the dipole and by forming a cavity mode, respectively. As seen from the representative optimized structure shown on the inset (orange star), we find that greater performance in this setting is achieved via a hybrid of the two single-material structures, highlighting that optimal structures may be combinations of single-material structures that leverage the benefits of both materials. As with the multilayer films, the bounds suggest that incorporating vacuum regions in metal-dielectric structures yields significant performance improvements while the inverse designs do not. In fact, optimization of heterostructures at *L*/*λ* = 1 showed the NV structure outperforming the V structure, highlighting the difficulty of optimizing heterostructures over more than two materials. This issue was remedied by initializing the V design problem with the local optimum of the NV design. This difficulty in optimization partially explains the non-zero duality gap but does not preclude the possible existence of tighter bounds.

Additionally, we consider the cases where *χ* ∈ {0, 4 + 0.1*i*, −4 + 1*i*, 8 + 0.2*i*} and *χ* ∈ {0, 4 + 0.1*i*, −4 + 1*i*, 8 + 1*i*}, where constraints are only enforced globally. The addition of a third, high-loss dielectric 8 + 1*i* to the two-material system does not alter the bound appreciably, demonstrating insensitivity to materials unable to produce larger resonant enhancements. This stands in contrast to the increased performance seen with a lower-loss material (8 + 0.2*i*), wherein the higher loss is compensated for by a corresponding increase in the index of refraction. To showcase the versatility of this method, a single bound is also calculated for *L*/*λ* = 0.3 and *χ* ∈ {0, 4 + 0.1*i*, −4 + 1*i*, −10 + 1*i*, −20 + 1*i*, −30 + 1*i*} (black cross), showing further albeit diminishing gains with larger number of materials.

## 5 Concluding remarks

This work shows that our extension to the duality bounds framework [7] for handling multi-material settings is effective and instructive. For the studied applications, access to multiple materials is shown to provide non-trivial performance benefits over single-material systems, except in very large designs where increased structural freedom makes greater material choice less consequential. While there is no proof of strong duality, the observation of optimized structures exceeding single-material bounds provides evidence that structures composed of multiple media offer meaningful advantages for photonic design. Results pertaining to metal-dielectric structures suggest that bounds may be used to assess the use of effective-medium theory in different application settings. Relatedly, results pertaining to dielectric heterostructures where effective medium theory precludes effective vacuum regions prove that the addition of vacuum in certain contexts can increase performance. Overall, promising applications of this theory include problems involving multiple sources that may be separately addressed by distinct material responses.

## Supplementary Material

Supplementary Material Details

## References

[1] Molesky S., Lin Z., Piggott A. Y., Jin W., Vucković J., Rodriguez A. W. (2018). Inverse design in nanophotonics. *Nat. Photonics*.

[2] Sitawarin C., Jin W., Lin Z., Rodriguez A. W. (2018). Inverse-designed photonic fibers and metasurfaces for nonlinear frequency conversion [invited]. *Photon. Res.*.

[3] Piggott A. Y., Lu J., Lagoudakis K. G., Petykiewicz J., Babinec T. M., Vučković J. (2015). Inverse design and demonstration of a compact and broadband on-chip wavelength demultiplexer. *Nat. Photonics*.

[4] Kao C. Y., Osher S., Yablonovitch E. (2005). Maximizing band gaps in two-dimensional photonic crystals by using level set methods. *Appl. Phys. B*.

[5] Angeris G., Vučković J., Boyd S. P. (2019). Computational bounds for photonic design. *ACS Photonics*.

[6] Molesky S., Chao P., Mohajan J., Reinhart W., Chi H., Rodriguez A. W. (2022). 𝕋-operator limits on optical communication: metaoptics, computation, and input-output transformations. *Phys. Rev. Res.*.

[7] Chao P., Strekha B., Defo R. K., Molesky S., Rodriguez A. W. (2022). Physical limits in electromagnetism. *Nat. Rev. Phys.*.

[8] Kuang Z., Miller O. D. (2020). Computational bounds to light–matter interactions via local conservation laws. *Phys. Rev. Lett.*.

[9] Gustafsson M., Schab K., Jelinek L., Capek M. (2020). Upper bounds on absorption and scattering. *New J. Phys.*.

[10] Molesky S., Jin W., Venkataram P. S., Rodriguez A. W. (2019). 𝕋operator bounds on angle-integrated absorption and thermal radiation for arbitrary objects. *Phys. Rev. Lett.*.

[11] Molesky S., Chao P., Jin W., Rodriguez A. W. (2020). Global 𝕋 operator bounds on electromagnetic scattering: upper bounds on far-field cross sections. *Phys. Rev. Res.*.

[12] Kuang Z., Miller O. D. (2022). Bounds on the coupling strengths of communication channels and their information capacities. *2022 Conference on Lasers and Electro-Optics (CLEO)*.

[13] Chao P., Defo R. K., Molesky S., Rodriguez A. (2023). Maximum electromagnetic local density of states via material structuring. *Nanophotonics*.

[14] Mohajan J., Chao P., Jin W., Molesky S., Rodriguez A. W. (2023). Fundamental limits on χ^(2)^ second harmonic generation. ..

[15] Gertler S., Kuang Z., Christie C., Miller O. D. (2023). Many physical design problems are sparse QCQPs. ..

[16] Yang C. (2016). Compact multilayer film structures for ultrabroadband, omnidirectional, and efficient absorption. *ACS Photonics*.

[17] Raman A. P., Anoma M. A., Zhu L., Rephaeli E., Fan S. (2014). Passive radiative cooling below ambient air temperature under direct sunlight. *Nature*.

[18] He J., Wang C., Zhou B., Zhao Y., Tao L., Zhang H. (2020). 2D van der Waals heterostructures: processing, optical properties and applications in ultrafast photonics. *Mater. Horiz.*.

[19] Lyu W. (2023). Fabrication and applications of heterostructure materials for broadband ultrafast photonics. *Adv. Opt. Mater.*.

[20] Soref R. (2014). Silicon-based silicon-germanium-tin heterostructure photonics. *Philos. Trans. Math. Phys. Eng. Sci.*.

[21] Miller O. D. (2023). Fundamental limits to near-field optical response. ..

[22] Luo Z.-Q., Ma W.-K., So A. M.-C., Ye Y., Zhang S. (2010). Semidefinite relaxation of quadratic optimization problems. *IEEE Signal Process. Mag.*.

[23] Boyd S., Vandenberghe L. (2004). *Convex Optimization*.

[24] ..

[25] Christiansen R. E., Sigmund O. (2021). Inverse design in photonics by topology optimization: tutorial. *J. Opt. Soc. Am. B*.

