# Supplementary material for: Can photonic heterostructures provably outperform single-material geometries?
Source: Nanophotonics. 2024 Jan 25;13(3):283–8. doi: 10.1515/nanoph-2023-0606 (PMC11501979; doi:10.1515/nanoph-2023-0606)
Supplement: Supplementary file 1 — Supplementary Material Details [file j_nanoph-2023-0606_suppl_001.pdf]

Supplemental Material for "Can photonic heterostructures provably outperform single-material geometries?"

Alessio Amaolo,[1] Pengning Chao,[2] Thomas J. Maldonado,[3] Sean Molesky,[4] and Alejandro W. Rodriguez[3]

[1]*Department of Chemistry, Princeton University, Princeton, New Jersey 08544, USA*
[2]*Department of Mathematics, Massachusetts Institute of Technology, Cambridge, Massachusetts 02139, USA*
[3]*Department of Electrical and Computer Engineering, Princeton University, Princeton, New Jersey 08544, USA*
[4]*Department of Engineering Physics, Polytechnique Montréal, Montréal, Québec H3T 1J4, Canada*

(Dated: December 13, 2023)

COMPUTING THE LAGRANGE DUAL OF THE MANY-MATERIAL PROBLEM

The primal optimization problem takes the form

$$\begin{aligned}
\max_{\{Tk,m\}} \quad & f(\{|T_{k,m}\rangle\}) \\
\text{s.t.} \quad & \sum_m \Big(\langle S_k| \mathbb{P}_j |T_{k',m}\rangle - \langle T_{k,m}| \chi_{k,m}^{-\dagger} \mathbb{P}_j |T_{k',m}\rangle - \sum_{m'} \langle T_{k,m}| - \mathbb{G}_0^{(k)\dagger} \mathbb{P}_j |T_{k',m'}\rangle \Big) = 0 \quad \forall j,k,k', \\
& \langle T_{k,m}| \mathbb{P}_j |T_{k',m'}\rangle = 0 \quad \forall j,k,k',m \neq m'.
\end{aligned} \tag{S1}$$

This notation differs from the main text via $\psi_{k,m} \to T_{k,m}$. As in the main text, $|S_k\rangle$ is a source k, the polarization current due to source k is $|T_k\rangle = \sum_m |T_{k,m}\rangle$ with $|T_{k,m}\rangle$ the polarization current due to source k and material m and is defined in the design region V. $\chi_{k,m}$ is the susceptibility of material m at ω_k, and $\mathbb{G}_0^{(k)}$ is the corresponding vacuum propagator acting on sources to yield their corresponding fields in vacuum—namely, via convolution of the vacuum Green's function $G_0^{(k)}(\mathbf{r},\mathbf{r}',\omega_k)$ satisfying $\frac{c^2}{\omega_k^2}\nabla\times\nabla\times G_0^{(k)}(\mathbf{r},\mathbf{r}',\omega_k) - G_0^{(k)}(\mathbf{r},\mathbf{r}',\omega_k) = \delta(\mathbf{r}-\mathbf{r}')$. $\mathbb{I}$ and $\mathbb{P}_j$ represent spatial projections onto either the full or a subset $V_j \in V$ of the design region V, respectively. Lastly, f is a quadratic function of the polarization currents $|T_{k,m}\rangle$.

For the first constraint, we will take the real and imaginary parts and write the Lagrange multiplier corresponding to a given j,k,k' as $\lambda_{R/I}^{j,k,k'}$ (symmetric and asymmetric constraints respectively). For the second, we will use Lagrange multipliers $\lambda_{SO/AO}^{k,k',m,m'}$ (symmetric/asymmetric orthogonal constraint). Now we can write

$$\mathcal{L}(T,S) = \begin{bmatrix} \langle T_{opt}| & \langle S| \end{bmatrix} \begin{bmatrix} -Z^{TT}(\lambda) & Z^{TS}(\lambda) \\ Z^{ST}(\lambda) & 0 \end{bmatrix} \begin{bmatrix} |T_{opt}\rangle \\ |S\rangle \end{bmatrix}, \tag{S2}$$

where $\mathcal{L}$ is the Lagrangian, $|T_{opt}\rangle = \begin{bmatrix} |T_{1,1}\rangle & |T_{1,2}\rangle & \dots & |T_{1,m}\rangle & \dots & |T_{2,1}\rangle & \dots & |T_{n_s,m}\rangle \end{bmatrix}^T$ for n_s sources and m materials, $|S\rangle = \begin{bmatrix} |S_1\rangle & \dots & |S_{n_s}\rangle \end{bmatrix}^T$, and Z^{TT} and $Z^{TS} = Z^{ST\dagger}$ matrices represent the quadratic and linear parts of the Lagrangian, respectively. We also denote N the numerical length of a single $|T_{k,m}\rangle$ vector.

Writing out the constraints we find

$$Z^{TS} = \mathbb{O}_{lin} + \sum_j \frac{1}{2} \begin{bmatrix} \begin{bmatrix} \lambda_R^{j,1,1}\mathbb{P}_j & \dots & \lambda_R^{j,n_s,1}\mathbb{P}_j \\ \vdots & \vdots & \vdots \\ \lambda_R^{j,1,1}\mathbb{P}_j & \dots & \lambda_R^{j,n_s,1}\mathbb{P}_j \end{bmatrix} \\ \vdots \\ \begin{bmatrix} \lambda_R^{j,1,n_s}\mathbb{P}_j & \dots & \lambda_R^{j,n_s,n_s}\mathbb{P}_j \\ \vdots & \vdots & \vdots \\ \lambda_R^{j,1,n_s}\mathbb{P}_j & \dots & \lambda_R^{j,n_s,n_s}\mathbb{P}_j \end{bmatrix} \end{bmatrix} - \sum_j \frac{1}{2i} \begin{bmatrix} \begin{bmatrix} \lambda_I^{j,1,1}\mathbb{P}_j & \dots & \lambda_I^{j,n_s,1}\mathbb{P}_j \\ \vdots & \vdots & \vdots \\ \lambda_I^{j,1,1}\mathbb{P}_j & \dots & \lambda_I^{j,n_s,1}\mathbb{P}_j \end{bmatrix} \\ \vdots \\ \begin{bmatrix} \lambda_I^{j,1,n_s}\mathbb{P}_j & \dots & \lambda_I^{j,n_s,n_s}\mathbb{P}_j \\ \vdots & \vdots & \vdots \\ \lambda_I^{j,1,n_s}\mathbb{P}_j & \dots & \lambda_I^{j,n_s,n_s}\mathbb{P}_j \end{bmatrix} \end{bmatrix}, \tag{S3}$$

$$Z^{ST} = Z^{TS\dagger}, \tag{S4}$$

where $\mathbb{O}_{lin}$ is the linear part of the objective.

$$Z^{TT} = \mathbb{O}_{quad} + \sum_j \begin{bmatrix} R^j_{1,1} & \dots & R^j_{1,n_s} \\ \vdots & \ddots & \vdots \\ R^j_{n_s,1} & \dots & R^j_{n_s,n_s} \end{bmatrix} + \sum_j \begin{bmatrix} I^j_{1,1} & \dots & I^j_{1,n_s} \\ \vdots & \ddots & \vdots \\ I^j_{n_s,1} & \dots & I^j_{n_s,n_s} \end{bmatrix} + \sum_j \begin{bmatrix} S^j_{1,1} + A^j_{1,1} & \dots & S^j_{1,n_s} + A^j_{1,n_s} \\ \vdots & \ddots & \vdots \\ S^j_{n_s,1} + A^j_{n_s,1} & \dots & S^j_{n_s,n_s} + A^j_{n_s,n_s} \end{bmatrix}, \tag{S5}$$

with

$$R^j_{k,k'} = R^{j\dagger}_{k',k} = \frac{1}{2}\lambda_R^{j,k,k'} \begin{bmatrix} (\chi_{k,1}^{-1\dagger}\mathbb{I} - \mathbb{G}_0^{(k)\dagger})\mathbb{P}_j & \dots & -\mathbb{G}_0^{(k)\dagger}\mathbb{P}_j \\ \vdots & \ddots & \vdots \\ -\mathbb{G}_0^{(k)\dagger}\mathbb{P}_j & \dots & (\chi_{k,m}^{-1\dagger}\mathbb{I} - \mathbb{G}_0^{(k)\dagger})\mathbb{P}_j \end{bmatrix} + \frac{1}{2}\lambda_R^{j,k',k} \begin{bmatrix} (\chi_{k',1}^{-1}\mathbb{I} - \mathbb{G}_0^{(k')})\mathbb{P}_j & \dots & -\mathbb{G}_0^{(k')}\mathbb{P}_j \\ \vdots & \ddots & \vdots \\ -\mathbb{G}_0^{(k')}\mathbb{P}_j & \dots & (\chi_{k',m}^{-1}\mathbb{I} - \mathbb{G}_0^{(k')})\mathbb{P}_j \end{bmatrix}, \tag{S6a}$$

$$I^j_{k,k'} = I^{j\dagger}_{k',k} = \frac{1}{2i}\lambda_I^{j,k,k'} \begin{bmatrix} (\chi_{k,1}^{-1\dagger}\mathbb{I} - \mathbb{G}_0^{(k)\dagger})\mathbb{P}_j & \dots & -\mathbb{G}_0^{(k)\dagger}\mathbb{P}_j \\ \vdots & \ddots & \vdots \\ -\mathbb{G}_0^{(k)\dagger}\mathbb{P}_j & \dots & (\chi_{k,m}^{-1\dagger}\mathbb{I} - \mathbb{G}_0^{(k)\dagger})\mathbb{P}_j \end{bmatrix} - \frac{1}{2i}\lambda_I^{j,k',k} \begin{bmatrix} (\chi_{k',1}^{-1}\mathbb{I} - \mathbb{G}_0^{(k')})\mathbb{P}_j & \dots & -\mathbb{G}_0^{(k')}\mathbb{P}_j \\ \vdots & \ddots & \vdots \\ -\mathbb{G}_0^{(k')}\mathbb{P}_j & \dots & (\chi_{k',m}^{-1}\mathbb{I} - \mathbb{G}_0^{(k')})\mathbb{P}_j \end{bmatrix}, \tag{S6b}$$

with $-\mathbb{G}_0^{(k)\dagger}\mathbb{P}_j$ or $-\mathbb{G}_0^{(k')}\mathbb{P}_j$ present in every off-diagonal element. Furthermore,

$$S^j_{k,k'} = \frac{1}{2}\begin{bmatrix} 0 & \mathbb{P}_j\lambda_{SO}^{j,k,k',1,2} & \dots & \mathbb{P}_j\lambda_{SO}^{j,k,k',1,m} \\ \mathbb{P}_j\lambda_{SO}^{j,k,k',1,2} & 0 & \dots & \mathbb{P}_j\lambda_{SO}^{j,k,k',2,m} \\ \vdots & \vdots & \ddots & \vdots \\ \mathbb{P}_j\lambda_{AO}^{j,k,k',1,m} & \mathbb{P}_j\lambda_{SO}^{j,k',2,m} & \dots & 0 \end{bmatrix} = {S^j_{k,k'}}^T = S^j_{k',k}, \tag{S7a}$$

$$A^j_{k,k'} = \frac{1}{2i}\begin{bmatrix} 0 & \mathbb{P}_j\lambda_{AO}^{j,k,k',1,2} & \dots & \mathbb{P}_j\lambda_{AO}^{j,k,k',1,m} \\ -\mathbb{P}_j\lambda_{AO}^{j,k,k',1,2} & 0 & \dots & \mathbb{P}_j\lambda_{AO}^{j,k,k',2,m} \\ \vdots & \vdots & \ddots & \vdots \\ -\mathbb{P}_j\lambda_{AO}^{j,k,k',1,m} & -\mathbb{P}_j\lambda_{AO}^{j,k,k',2,m} & \dots & 0 \end{bmatrix} = {A^j_{k,k'}}^\dagger = -A^j_{k',k}. \tag{S7b}$$

(S7c)

$\mathbb{O}_{quad}$ is the quadratic part of the objective and all R, I, S, A are $m \times m$ block matrices of $N \times N$ matrices. We can compute the dual function $\mathcal{G}$:

$$\mathcal{G}(\lambda) = \sup_{|T\rangle} \mathcal{L}(\lambda, T). \tag{S8}$$

We find the stationary point $|T^*\rangle$ of $\mathcal{L}$ by solving the relation

$$\frac{\partial \mathcal{L}}{\partial \langle T^*|} = 0, \tag{S9}$$

which leads to the linear system

$$Z^{TT}|T^*\rangle = Z^{TS}|S\rangle. \tag{S10}$$

In order for the dual to be finite, Z^{TT} must be positive definite, so this linear system is invertible, leading to

$$|T^*\rangle = Z^{TT-1}Z^{TS}|S\rangle, \tag{S11}$$

$$\mathcal{G}(\lambda) = \langle S| Z^{ST}Z^{TT-1}Z^{TS}|S\rangle. \tag{S12}$$

Finally,

$$\frac{\partial \mathcal{G}}{\partial \lambda_i} = 2\,\mathrm{Re}\left\{\langle T^*|\frac{\partial Z^{TS}}{\partial \lambda_i}|S\rangle\right\} - \langle T^*|\frac{\partial Z^{TT}}{\partial \lambda_i}|T^*\rangle. \tag{S13}$$

In the specific case of maximizing absorption, $\mathbb{O}_{lin} = 0$. Absorbed power is

$$\sum_{k,m} \frac{Zc}{2\omega_k}\langle T_{k,m}|\frac{\mathrm{Im}\,\chi_{k,m}}{|\chi_{k,m}|^2}|T_{k,m}\rangle, \tag{S14}$$

with Z the vacuum impedance, giving us $\mathbb{O}_{quad}$ (being careful of the negative sign in Eq. (S2)). Each term can be normalized as desired by the incident or total power.

SOLVING THE DUAL PROBLEM

In order to solve the convex dual problem using an interior point method, we must first find an initial feasible point. This requires choosing λ such that Z^{TT} is positive definite, thereby ensuring that Z^{TT} is invertible and therefore that the dual is well defined. This can be done reliably by leveraging the fact that the imaginary part of the Maxwell Green's function, $\mathrm{Im}\,\mathbb{G}_0^{(k)}$, is positive semidefinite [4]. Take $\mathbb{P}_{j=0} = \mathbb{I}$ to be the projector corresponding to global constraints, which is always enforced in our implementation. By setting all Lagrange multipliers $\lambda = 0$ except $\lambda_I^{j=0,k,k}$

for all k, we set $R^j_{k,k'} = S^j_{k,k'} = A^j_{k,k'} = 0$ for all k, k', j. This also sets $I^j_{k,k'} = 0$ for all $j, k \neq k'$ and, although not necessary, for all $j \neq 0, k = k'$. The quadratic part of $\mathcal{L}$ becomes

$$Z^{TT} = \mathbb{O}_{quad} + \begin{bmatrix} I^0_{1,1} & \dots & 0 \\ \vdots & \ddots & \vdots \\ 0 & \dots & I^0_{n_s,n_s} \end{bmatrix}, \tag{S15}$$

with $I^0_{k,k}$ taking the following block diagonal form:

$$\begin{aligned} I^0_{k,k} &= \lambda_I^{0,k,k} \begin{bmatrix} \mathrm{Asym}\left(\mathbb{I}\chi^{-1\dagger}_{k,1} - \mathbb{G}_0^{(k)\dagger}\right) & \dots & 0 \\ \vdots & \ddots & \vdots \\ 0 & \dots & \mathrm{Asym}\left(\mathbb{I}\chi^{-1\dagger}_{k,m} - \mathbb{G}_0^{(k)\dagger}\right) \end{bmatrix} \\ &= \lambda_I^{0,k,k} \begin{bmatrix} \mathbb{I}\frac{\mathrm{Im}\,\chi_{k,1}}{|\chi_{k,1}|^2} + \mathrm{Asym}\mathbb{G}_0^{(k)} & \dots & 0 \\ \vdots & \ddots & \vdots \\ 0 & \dots & \mathbb{I}\frac{\mathrm{Im}\,\chi_{k,1}}{|\chi_{k,m}|^2} + \mathrm{Asym}\mathbb{G}_0^{(k)} \end{bmatrix}, \end{aligned} \tag{S16}$$

which is positive definite for $\mathrm{Im}\,\chi_{k,m} > 0$. Therefore, as long as all materials have some loss (as they do in the main text), we can simply increase $\lambda_I^{0,k,k}$ for all k to make $Z^{TT}(\lambda)$ positive definite for any finite $\mathbb{O}_{quad}$. We initialize all bounds calculations in the main text (where $n_s = 1$) by setting $\lambda_I^{0,k,k} = 1 \quad \forall k$. When $\mathrm{Im}\,\chi_{k,m} = 0$, we may be able to generalize the technique utilized in Ref [1] (Supporting Information, Section 11).

DESIGN REGIONS WITH NON-VACUUM BACKGROUNDS

The calculations in the previous section assumed the design region has vacuum background. However, the scattering theory can be readily modified to include non-vacuum backgrounds. We define χ_b as the background susceptibility, $\Delta\chi_{k,m} \equiv \chi_{k,m} - \chi_b$, and write the total field at at frequency k as $E_{k,tot}$. We also define the difference in polarization $|\Delta T_{k,m}\rangle \equiv |T_{k,m}\rangle - |T_b\rangle \sim \Delta\chi_{k,m}E_{k,tot}$ where $|T_b\rangle$ is the polarization of the background. The Green's function including the background $\mathbb{G}_b^{(k)}$ is defined to map a difference in polarization to its resulting field: $\mathbb{G}_b^{(k)}|\Delta T_{k,m}\rangle$ is the scattered field due to the additional polarization. Lastly, assuming $|S_k\rangle$ is sourced by some current J_{vac}, we replace $|S_k\rangle \to |S_{k,b}\rangle$ by the field sourced by the same J_{vac} in the presence of the background design region. Overall, the scattering theory is defined by the relations $|E_{k,tot}\rangle \sim |S_{k,b}\rangle + \sum_m \mathbb{G}_b^{(k)}|\Delta T_{k,m}\rangle$. The new optimization problem can be written

$$\begin{aligned} \max_{\{\Delta T_{k,m}\}} \quad & f(\{|T_b\rangle, |\Delta T_{k,m}\rangle\}) \\ \text{s.t.} \quad & \sum_m \left(\langle S_{k,b}|\mathbb{P}_j|\Delta T_{k',m}\rangle - \langle \Delta T_{k,m}|\Delta\chi^{-\dagger}_{k,m}\mathbb{P}_j|\Delta T_{k',m}\rangle - \sum_{m'}\langle \Delta T_{k,m}| - \mathbb{G}_b^{(k)\dagger}\mathbb{P}_j|\Delta T_{k',m'}\rangle \right) = 0 \quad \forall j,k,k', \\ & \langle \Delta T_{k,m}|\mathbb{P}_j|\Delta T_{k',m'}\rangle = 0 \quad \forall j,k,k',m \neq m'. \end{aligned} \tag{S17}$$

where f must be modified accordingly by replacement of $|T_{k,m}\rangle \to \Delta T_{k,m} + |T_b\rangle$.

NUMERICAL DETAILS: SPARSE FORMULATION

The optimization problem described in Eq. S1 contains a very large number of constraints, making the calculation of Z^{TT} computationally expensive. However, noting that $\mathbb{G}_0$ is proportional to the inverse of the sparse Maxwell

operator $\mathbb{M}$, we can rewrite the constraints in Eq. S1

$$\sum_m \Big(\langle S_k| \mathbb{P}_j \mathbb{G}_0^{-(k')} \mathbb{G}_0^{(k')} |T_{k',m}\rangle - \langle T_{k,m}| \mathbb{G}_0^{(k)\dagger} \mathbb{G}_0^{-(k)\dagger} \mathbb{P}_j \chi_{k,m}^{-\dagger} \mathbb{G}_0^{-(k')} \mathbb{G}_0^{(k')} |T_{k',m}\rangle$$
$$- \sum_{m'} \langle T_{k,m}| \mathbb{G}_0^{(k)\dagger} \mathbb{G}_0^{-(k)\dagger} (-\mathbb{G}_0^{(k)\dagger}) \mathbb{P}_j G_0^{-(k')} \mathbb{G}_0^{(k')} |T_{k',m'}\rangle \Big) = 0 \quad \forall j, k, k', \tag{S18}$$
$$\langle T_{k,m}| \mathbb{G}_0^{(k)\dagger} \mathbb{G}_0^{-(k)\dagger} \mathbb{P}_j G_0^{-(k')} \mathbb{G}_0^{(k')} |T_{k',m'}\rangle = 0 \quad \forall j, k, k', m \neq m'.$$

Now, taking $\mathbb{G}_0^{(k)} |T_{k,m}\rangle$ as the new optimization vector and cancelling inverses, all constraints can be represented using sparse matrices. $\mathbb{G}_0^{-1}$ can be calculated using Woodbury inversion of known Maxwell operators.

INVERSE DESIGN DETAILS

All calculations were run at increasing resolutions until converged. Multiple material topology optimization was done by writing $\epsilon = \epsilon_2 + (\epsilon_1 + (\epsilon_{\text{background}} - \epsilon_1)\rho_2 - \epsilon_2)\rho_1$ for $\rho_1, \rho_2 \in [0, 1]$ and optimizing over the continuous variables ρ_1, ρ_2. The derivatives of the objective with respect to modifications in ρ_1, ρ_2 were computed with Ceviche [2]. The resulting optimization problem was solved with NLopt [3].

Inverse designs are often binarized to reflect realistic devices. In the multi-material case, we define a binarized design as one where each pixel is exclusively one of the available materials. To better compare with bounds (which at high enough resolutions mimic the behavior of non-binarized devices), inverse designs were not deliberately binarized with the exception of when vacuum is not available in the optimization problem. In these examples shown, binarization only marginally affected performance.

[1] Pengning Chao, Rodrick Kuate Defo, Sean Molesky, and Alejandro Rodriguez. Maximum electromagnetic local density of states via material structuring. *Nanophotonics*, 12(3):549–557, 2 2023.

[2] Tyler W Hughes, Ian AD Williamson, Momchil Minkov, and Shanhui Fan. Forward-mode differentiation of maxwell's equations. *ACS Photonics*, 6(11):3010–3016, 2019.

[3] Steven G. Johnson. The NLopt nonlinear-optimization package. `https://github.com/stevengj/nlopt`, 2007.

[4] Leung Tsang, Jin Au Kong, and Kung-Hau Ding. *Scattering of Electromagnetic Waves: Theories and Applications*, volume 27. John Wiley & Sons, 2004.
